# Self-Perceived Knowledge, Self-Efficacy, and Reported Integration of Generative Artificial Intelligence Among University Faculty: An Exploratory Profile Analysis

**DOI:** 10.3390/bs16101878

**Published:** 2026-10-12

**Authors:** Graciela Castro-Castillo, Víctor Bosquez-Barcenes, Mariuxi Vinueza-Morales, Cristian Vidal-Silva

**Affiliations:** 1 Facultad de Posgrado, Universidad Estatal de Milagro (UNEMI), Milagro 091050, Ecuador; gcastroc4@unemi.edu.ec; 2 Facultad de Ciencias e Ingeniería, Universidad Estatal de Milagro (UNEMI), Milagro 091050, Ecuador; 3 Facultad de Ingeniería y Negocios, Universidad de Las Américas, Manuel Montt 948, Providencia, Santiago 7500975, Chile

**Keywords:** generative artificial intelligence, teacher training, self-efficacy, pedagogical integration, higher education, teacher profiles

## Abstract

Generative artificial intelligence (GAI) adoption in higher education raises questions about the relationships among perceived knowledge, confidence, attitudes, and reported teaching practices. A cross-sectional study of 149 faculty members at an Ecuadorian public university examines four self-reported constructs, with Cronbach’s α ranging from 0.841 to 0.981. An exploratory three-group partition describes higher-scoring (44.3%), intermediate-scoring (39.6%), and lower-scoring (16.1%) respondents, although the silhouette criterion favors two groups (0.383 versus 0.299). Self-efficacy correlates with reported integration (r=0.812; approximate 95% CI [0.749, 0.860]). A complementary three-predictor regression reconstructed from the reported correlation matrix yields $R^{2}=0.668$ and a self-efficacy coefficient of β=0.758 (approximate 95% CI [0.630, 0.885]). This model excludes demographic and frequency covariates and does not replace the original adjusted analysis. Reconstructed aggregate counts show an association between profile and grouped use frequency (conditional Monte Carlo p=0.0037), whereas the gender association remains inconclusive (p=0.089). Measurement overlap, self-reporting, and unverified cluster stability limit interpretation. Normalizing response ranges does not establish that faculty practice more than they know. The results support evaluating faculty development that combines conceptual and critical AI literacy with GAI-specific self-efficacy; causal effectiveness remains untested.

## 1. Introduction

The availability of conversational generative artificial intelligence (GAI) systems has prompted renewed discussion of teaching, assessment, and faculty preparation. Educational opportunities coexist with concerns about unreliable outputs, academic integrity, and responsible use (García-Peñalvo, 2023; Kasneci et al., 2023). Understanding faculty engagement requires distinguishing what instructors believe they understand, what they feel able to do, and what they report doing. These dimensions are related but neither interchangeable nor direct measures of pedagogical quality.

Technological pedagogical content knowledge provides a framework for examining the relationship between technology, teaching, and disciplinary expertise. Its reconsideration in the context of GAI highlights the need to situate technological use within educational purposes (Mishra et al., 2023). Intelligent-TPACK additionally addresses professional knowledge for ethical integration of AI-based tools (Celik, 2023). These frameworks motivate the joint consideration of knowledge and practice; they do not justify subtracting scores from instruments measuring different constructs.

The present study examines four self-reported dimensions among faculty in an Ecuadorian faculty of education: perceived conceptual knowledge, reported pedagogical integration, perceived self-efficacy, and attitudes. Its objectives are to describe their associations, explore a descriptive partition of respondents, and examine adjusted associations with reported integration. The design does not test whether training causes adoption or whether confidence mediates a knowledge–practice relationship.

### 1.1. GAI in Higher Education and the Scope of Prior Evidence

Research on GAI encompasses student perceptions, teacher readiness, and conceptual discussions of educational change. Chan and Hu (2023) examine university students’ perceptions, benefits, and concerns. Moorhouse (2024) explores readiness among beginning and first-year language teachers through interviews. Lim et al. (2023) analyze educational tensions through paradox theory. These contributions address complementary questions, but their populations and methods differ from a quantitative survey restricted to university faculty.

Recent student-level evidence also broadens the educational context. Rehman and colleagues survey 813 non-native university students at seven institutions in China and use structural equation modeling to examine associations among GAI affordances, cognitive load reduction, academic resilience, and personalized learning (Rehman et al., 2026). Their cross-sectional findings motivate attention to learning-related experiences, but do not establish causal effects, historically unprecedented adoption, or the competence of university instructors. The present faculty survey addresses a different population and does not measure cognitive load or student learning.

A systematic review in *Behavioral Sciences* distinguishes responses, attitudes, and utilization behaviors in higher education (Wu et al., 2025). This distinction is useful for the present study: favorable views, confidence, and reported use are separate dimensions even when they correlate. A faculty member may endorse educational opportunities without reporting frequent implementation, while frequent implementation does not by itself demonstrate an ability to evaluate the quality of generated material.

Ecuadorian evidence on broader digital teaching competence also provides relevant context. Moreira-Choez et al. (2024) examine self-perceived digital competence among professors at an Ecuadorian university. Their study concerns general digital teaching competence rather than the four GAI constructs examined here. It supports the relevance of a context-specific faculty perspective while underscoring that self-perception should not be equated with independently demonstrated proficiency.

These distinctions matter when motivating faculty research. Evidence about student perceptions, teacher readiness, and conceptual tensions can inform research questions without supplying direct estimates for the present population. UNESCO guidance and its teacher competency framework provide complementary policy perspectives on responsible educational engagement with AI (Miao & Cukurova, 2024; Miao & Holmes, 2023). Such guidance supports attention to educational purposes and ethical understanding, rather than interpreting frequent use alone as successful integration.

Broader reviews distinguish applications of AI in higher education from evidence about how teachers use those applications. Crompton and Burke map the higher-education research field, while Celik and colleagues review promises and challenges specifically relevant to teachers (Celik et al., 2022; Crompton & Burke, 2023). Grassini discusses the educational opportunities and consequences of AI and conversational generative AI tools, and Flores-Vivar and García-Peñalvo examine ethical considerations in relation to quality education (Flores-Vivar & García-Peñalvo, 2023; Grassini, 2023). Together, these contributions motivate examining both educational use and the understanding needed to appraise it, without treating adoption as an outcome measure of teaching quality.

Academic-integrity concerns also require careful attribution. Sullivan and colleagues analyze media coverage of ChatGPT and higher education; their evidence concerns public discussion and institutional responses rather than a direct survey of faculty competence (Sullivan et al., 2023). In Latin American higher education, Vargas Bernuy and colleagues study ChatGPT adoption through relative advantage and compatibility (Vargas Bernuy et al., 2025). Such adoption models complement, but do not replace, an assessment of perceived knowledge, self-efficacy, attitudes, and reported integration within a specific faculty.

### 1.2. Perceived Knowledge and Reported Practice

Perceived conceptual knowledge concerns respondents’ assessments of their understanding. Reported pedagogical integration concerns their accounts of teaching-related uses. An objective knowledge test, an observed classroom practice, and a self-report rating provide different kinds of evidence. Accordingly, the present questionnaire cannot determine whether respondents’ confidence in their understanding corresponds to demonstrated competence.

Different response ranges create an additional interpretive issue. A theoretical min–max transformation maps each scale onto 0–1 but preserves its original construct and measurement limitations. Equal transformed values do not establish equal mastery, equal difficulty, or equivalent distances between substantive levels. The analysis therefore emphasizes associations and within-construct differences across groups. It does not interpret cross-construct score differences as a deficit in knowledge relative to practice.

### 1.3. Self-Efficacy and Measurement Overlap

Bandura’s account of self-efficacy provides the theoretical basis for distinguishing perceived capability from demonstrated performance (Bandura, 1977). Evidence about teacher preparation spans several related domains. Hu and colleagues review self-efficacy and digital competence, whereas Blannin and colleagues examine teacher self-efficacy to teach digital technologies in Australian schools (Blannin et al., 2026; Hu et al., 2025). These studies motivate attention to confidence in educational technology, but general digital competence and school teaching are not identical to GAI integration among university faculty.

More directly, Ofori Darko and colleagues examine teachers’ competence, self-efficacy, and attitudes toward generative AI in a correlational study (Ofori Darko et al., 2026). Alvarez and colleagues distinguish future teachers’ beliefs as students and as aspiring professionals, while Kul and colleagues investigate pre-service teachers’ TPACK and generative-AI acceptance (Alvarez et al., 2024; Kul et al., 2026). Their different professional roles and constructs argue for specifying the activity and population to which a self-efficacy interpretation applies. Taheri and colleagues review factors influencing educators’ AI adoption, providing a broader synthesis of adoption conditions rather than a direct estimate for the present sample (Taheri et al., 2025).

The literature on outcomes and interventions addresses different questions from those answered here. Ma and colleagues synthesize associations between teacher self-efficacy and student academic performance; Täschner and colleagues review interventions intended to promote teacher self-efficacy (Ma et al., 2025; Täschner et al., 2025). Liu and colleagues examine generative-AI self-efficacy and reflection in relation to second-language achievement (Liu et al., 2025). These contributions help motivate future outcome-based studies, but no student achievement measure or intervention is included in the present design.

Self-efficacy is conceptually distinct from reported use: feeling able to perform an activity need not entail performing it. Nevertheless, questionnaire items referring to similar activities can produce overlapping measures. Scherer et al. (2020) examine the measurement structure of teachers’ technology acceptance and provide a relevant methodological context for scrutinizing distinctions among correlated beliefs and reported use. Their work highlights the importance of examining whether correlated measures capture distinguishable aspects of technology acceptance.

The present scales include closely related content, such as creating teaching activities, integrating GAI into workflows, and evaluating generated material. A strong association between their composite scores may consequently reflect substantive relationships, shared wording or content, and common response processes. Internal consistency alone cannot distinguish these explanations (Sijtsma, 2009). Shared measurement context can also affect associations among self-reported constructs (Podsakoff et al., 2003). The regression is interpreted as an exploratory analysis of adjusted associations, conditional on these measurement limitations.

### 1.4. An Exploratory Person-Centered Perspective

A profile analysis can complement correlations by describing combinations of responses within a sample. However, a clustering algorithm partitions observations even when the underlying variation is continuous. A selected solution therefore requires evaluation of separation, stability, preprocessing choices, and interpretability before it can support claims about distinct underlying types. Model-based clustering offers alternative approaches to this problem (Scrucca et al., 2024). The silhouette evaluates cohesion and separation of a partition; it does not establish a substantive theory of faculty types (Rousseeuw, 1987).

Recent applications illustrate the range of questions addressed by person-centered analyses. Adamoli and colleagues investigate cluster-based profiles of social educators and teachers in Italy, and Saihi and Ahmed examine adoption personas for generative AI in higher education (Adamoli et al., 2026; Saihi & Ahmed, 2026). García-Ortiz and colleagues use TPACK-based teacher profiling for educational-technology recommendations, while Chen and colleagues apply latent profile analysis to pre-service teachers’ ChatGPT acceptance across STEM and non-STEM disciplines (Chen et al., 2026; García-Ortiz et al., 2026). These studies provide methodological and contextual precedents, not external validation of the groups found here. Cluster stability is a distinct empirical question that requires explicit evaluation, for example through resampling-based assessments (Hennig, 2007).

Here, the three-group partition is retained only as a descriptive account of the reported analysis. Neutral labels refer to comparatively higher, intermediate, or lower scores within this sample. They do not imply expertise, reluctance, critical judgment, or pedagogical risk. The stronger silhouette value for two clusters remains a substantive qualification of the three-group presentation.

Three research questions guide the analysis: (1) How are the four self-report composites associated? (2) What descriptive patterns are present in the reported clustering, and how do their external characteristics differ? (3) How does the association of perceived knowledge with integration change when self-efficacy and attitudes are considered jointly? Supplementary calculations from aggregate statistics assess uncertainty and selected reporting checks. They do not reconstruct respondent-level answers.

## 2. Materials and Methods

### 2.1. Study Design and Participants

A quantitative, non-experimental, cross-sectional study examines associations among four self-report composites: perceived conceptual knowledge of GAI, reported pedagogical integration, perceived self-efficacy, and attitudes. The analytical strategy follows the three research questions. First, descriptive statistics and Pearson correlations characterize the composites and their bivariate associations. Second, the reported *k*-means partition describes within-sample response patterns, with exploratory comparisons of selected participant characteristics. Third, hierarchical regression examines adjusted associations with reported integration. A complementary regression reconstructed from aggregate correlations provides a transparent numerical assessment under explicitly stated assumptions. Neither analytical approach establishes temporal order, causal effects, or validated faculty types.

The sample consisted of 149 faculty members of the Faculty of Education Sciences (FACE) at a public university on the coast of Ecuador. Census sampling was used: the entire active faculty of the school was invited to participate, with a response rate of 89.2%. Of the total, 105 participants (70.5%) identified as women and 44 (29.5%) as men. Age ranged from 24 to 65 years (M=42.5, SD=10.1), and teaching experience from 1 to 48 years (M=14.1, SD=10.0). Regarding highest academic qualification, 103 (69.1%) held a master’s degree, 20 (13.4%) a doctorate, 22 (14.8%) a bachelor’s degree, 2 a postdoctoral qualification, and 2 a specialization diploma. Participants came from programs delivered online (45.0%), in person (28.2%), and in blended format (15.4%), with 11.4% missing modality data.

Frequency of GAI use varied: 72 faculty members (48.3%) reported daily use, 61 (40.9%) weekly use, 13 (8.7%) occasional use (less than once a month), and 3 (2.0%) a single trial use. Training-recency counts sum to 147 responses, rather than the full sample of 149. The original report labels 65 responses (44.2%) “within the last year”, 48 (32.7%) “within the last six months”, 19 (12.9%) “within the last five years”, 8 (5.4%) “more than five years ago”, and 7 (4.8%) “never”. These labels overlap and cannot be interpreted as mutually exclusive intervals without checking the questionnaire.

Table 1 summarizes the principal participant characteristics. Counts for teaching modality are uniquely reconstructed from the reported one-decimal percentages and N=149; other counts are supplied directly in the original report. Percentages are calculated within each indicated denominator.

### 2.2. Instruments

An ad hoc questionnaire organized into five sections was designed. The first section collected sociodemographic and professional data (gender, age, teaching experience, program, courses taught, academic qualification, training in educational technologies, and frequency of GAI use). The remaining four sections corresponded to the study’s core constructs.

*Self-perceived conceptual knowledge of GAI* (12 items, 7-point Likert scale) assessed self-reported understanding of how language models work, the distinction between GAI and search engines, the concept of prompts, the identification of hallucinations and biases, the concepts of tokens, context, and temperature, the distinction among types of GAI, and the ethical implications of educational use. Three items were reverse-worded to address acquiescent responding and recoded before computing the composite score. Internal consistency was adequate (α=0.841).

*Pedagogical integration of GAI* (15 items, 5-point Likert scale) assessed declared teaching practices, including designing personalized activities with GAI, creating educational prompts, methodological adaptation, designing ethical assessments, teaching students to formulate effective questions, using GAI for personalized feedback, developing rubrics, creating collaborative scenarios, differentiating instruction, developing critical thinking about outputs, verifying generated information, and modeling responsible use. Internal consistency was excellent (α=0.981), a value that warrants a note of caution: alphas above 0.95 can indicate item redundancy or common-method variance, an issue revisited in Section 4.4.

*Perceived self-efficacy for GAI* (10 items, 5-point Likert scale) measured perceived confidence in solving technical problems with GAI, teaching colleagues, identifying incorrect information, creating activities in under 30 minutes, guiding ethical student use, evaluating generated content, customizing settings, integrating GAI into one’s teaching workflow, troubleshooting students’ difficulties with the tool, and staying up to date. Internal consistency was excellent (α=0.947).

*Attitudes toward GAI in the classroom* (8 items, 7-point semantic differential) presented pairs of opposing adjectives referring to GAI in the classroom. Internal consistency was excellent (α=0.949).

The instrument description does not document objectively scored knowledge questions or independent observations of teaching. Consequently, knowledge and integration are interpreted throughout as self-perceived knowledge and self-reported integration, respectively. Cronbach’s alpha describes internal consistency; it does not establish factorial or discriminant validity. The very high integration alpha and overlap with self-efficacy require particular caution.

Scale development involves specifying content, evaluating items, and examining dimensionality and validity, beyond reporting a reliability coefficient (Boateng et al., 2018). These stages provide a framework for the additional measurement work required here; their citation does not imply that undocumented procedures were performed.

Table 2 summarizes the reported content of the instrument. It is a construct map rather than a reproduction of the administered questionnaire. Exact item wording, response anchors, expert-review records, and pilot documentation are unavailable in the materials supporting this revision. Consequently, no additional validation procedures are claimed. Expert review and pilot testing, if performed, require their original records. Exploratory or confirmatory factor analysis and convergent or discriminant validity assessment require item-level data and an explicit measurement model; none can be recovered from four composite correlations. The four scores are therefore treated as provisional operational composites, not established measures of four empirically distinct latent constructs. Reproducibility also requires the exact administered language, item order, response anchors, reverse-scoring key, and composite-scoring rules. The content descriptions in Table 2 cannot establish how particular wording influenced responses. An accessible archived copy of the administered questionnaire is needed before independent replication; no public questionnaire deposit is documented in the available materials.

### 2.3. Procedure

The instrument was administered via Google Forms between November 2025 and August 2026. Participants received an invitation through their institutional email with a description of the study, its objectives, and a guarantee of data confidentiality. Participation was voluntary, and digital informed consent was obtained prior to completing the questionnaire. Average response time was approximately 15 min. Data were exported to spreadsheet format for processing.

### 2.4. Statistical Analysis

The analysis distinguishes original reported results from complementary calculations using aggregate statistics. The original study reports descriptive statistics, Shapiro–Wilk tests, Pearson correlations, *k*-means clustering, profile comparisons, and hierarchical regression, implemented with Python 3.4, pandas 0.20.3, SciPy 1.2.0, scikit-learn 0.20.4, and statsmodels 0.8.0. The available analytical record contains composite means, standard deviations, correlations, profile summaries, and regression coefficients, but not individual responses or the original executable analysis. Newly derived values are therefore explicitly identified as approximate or reconstructed.

#### 2.4.1. Scale Interpretation and Uncertainty

Theoretical min–max scores, u=(x−L)/(U−L), describe position within a response range. They differ from *z* scores, $z=(x-\bar{x})/s$, which describe deviation from a sample mean. Neither makes two constructs psychometrically equivalent. Cross-construct paired tests are therefore not used to infer a knowledge–practice deficit. Descriptive profile figures retain the original response scales. The distinction also matters for Euclidean-distance clustering: separate min–max and *z* transformations generally weight variables differently. If the same nonconstant variable and the same observations are used, a positive affine transformation followed by *z* standardization satisfies z(ax+b)=z(x) for a>0. This algebraic identity clarifies one possible sequence; it does not identify which sequence was implemented in the source analysis. No preprocessing sequence is retroactively assigned without the original code.

Approximate 95% confidence intervals for full-sample means use $\bar{x}\pm t_{0.975,148}s/\sqrt{149}$. Correlation intervals use the Fisher transformation with standard error $1/\sqrt{149-3}$, applying a normal 1.96 critical value before back-transformation. These are conventional model-based intervals from rounded summaries; they are neither bootstrap intervals nor distribution-robust estimates. They assume independent observations and an appropriate sampling model. Given the census invitation and nonresponse, they should not be read as design-based intervals for the institution’s finite faculty population. Reported departures from normality further warrant caution about nominal coverage.

#### 2.4.2. Exploratory Profiles and Reconstructed Counts

The original clustering considers k=2 through k=6, although the available analytical record provides silhouette coefficients only for k=2 and k=3. The reported coefficients favor two groups (0.383 versus 0.299). Accordingly, the three-group partition is presented as a descriptive summary of the previously reported solution, rather than as the statistically preferred number of clusters. Retaining this partition preserves the available group summaries; it does not establish an additional intermediate faculty type. Respondent-level data would be required to recover the two-group memberships, compare the partitions, and evaluate stability. The original wording combines normalization and standardization without an unambiguous implementation record. Consequently, the actual preprocessing sequence, initialization, and stability cannot be verified. Significance tests on the variables used to create the groups are not treated as independent validation. Specifically, ANOVA and Kruskal–Wallis tests on the four clustering composites are omitted from the revised results. Group construction and group comparison use the same information, so ordinary post-clustering significance does not independently establish meaningful profiles. Comparisons involving gender or use frequency are exploratory external descriptions, conditional on the selected partition; they do not establish cluster stability.

Gender counts and daily/weekly-use counts are uniquely recoverable from the profile sizes and one-decimal percentages. For each cell, the integer count is required to reproduce the displayed percentage within its rounding interval. Subtracting daily and weekly counts from profile totals yields the combined occasional/single-trial category. This aggregation does not identify the original two low-frequency categories separately.

For gender, a Pearson chi-square test and Cramér’s *V* are calculated from the reconstructed 3×2 table. For the grouped frequency table, one expected count is below five; a conditional Monte Carlo test uses 100,000 random contingency tables with the observed margins, Pearson’s statistic, and seed 4,570,013. The tail probability is computed as (b+1)/(100,000+1). These simulations represent a null distribution, not simulated respondents. All profile-characteristic analyses are exploratory, with no familywise multiplicity adjustment.

#### 2.4.3. Original and Complementary Regression Models

The original hierarchical model enters age, experience, gender, and use frequency at Step 1; perceived knowledge at Step 2; and self-efficacy and attitudes at Step 3. The frequency coding and exact analytic sample at each step are undocumented. Approximate normal-reference intervals, B±1.96SE, are added to its reported coefficients without asserting exact model degrees of freedom or correcting the original *p*-values. The notation *B* is retained from the source and is not relabeled as standardized. Its status as standardized or unstandardized cannot be confirmed from the supplied model output; consequently, coefficient magnitudes are not used for cross-predictor comparisons in that model. Standardized coefficients are explicitly calculated only for the complementary model below.

A separate, complementary ordinary least-squares model is reconstructed from the four-construct correlation matrix, assuming its stated N=149 represents the same complete observations for every correlation. Reported integration is the outcome; perceived knowledge, self-efficacy, and attitudes are the three predictors. With predictor correlation matrix $R_{XX}$ and predictor–outcome vector $r_{Xy}$,

(1) $$\hat{\beta }=R_{XX}^{-1}r_{Xy},\;R^{2}=r_{Xy}^{T}\hat{\beta },\;\mathrm{SE}\left({\hat{\beta }}_{j}\right)=\sqrt{\frac{1-R^{2}}{149-3-1}{\left(R_{XX}^{-1}\right)}_{jj}}.$$

Approximate coefficient intervals use a *t* reference with 145 degrees of freedom. Unstandardized coefficients follow $B_{j}=\beta_{j}s_{y}/s_{j}$, and predictor VIFs equal the diagonal elements of $R_{XX}^{-1}$. These conventional OLS calculations do not provide heteroskedasticity-robust inference or residual diagnostics. They exclude the demographic and frequency covariates and cannot substitute for re-estimation of the original seven-predictor model.

To examine rounding sensitivity, the six off-diagonal correlations are each perturbed by ±0.0005, producing 64 corner matrices. All are checked for positive definiteness. The observed ranges across these evaluated matrices describe numerical sensitivity; they are not confidence intervals, empirical replications, or guaranteed global bounds over the rounding region.

### 2.5. Ethical Considerations

Participation is described in the source manuscript as voluntary, with digital informed consent, anonymization of institutional email addresses before analysis, and storage on institutional servers restricted to the research team. The reported collection period is November 2025–August 2026. The approval code, committee identification, and date remain to be verified against the original ethics decision before submission; the reported collection dates must be covered by that decision.

## 3. Results

### 3.1. Descriptive Statistics and Reliability

Table 3 presents the reported descriptive statistics. The mean is 5.00 on the 1–7 perceived-knowledge scale, 3.93 on the 1–5 integration scale, 3.99 on the 1–5 self-efficacy scale, and 5.63 on the 1–7 attitudes scale. All exceed their respective response-scale midpoints, without establishing substantive mastery thresholds. Distributions are negatively skewed, and the reported Shapiro–Wilk tests reject normality for all four composites (p<0.01). The observed ranges reported in the base manuscript are 1.67–7.00 for knowledge, 1.00–5.00 for integration and self-efficacy, and 2.25–7.00 for attitudes. Distributional non-normality motivates robustness checks; it does not by itself determine whether a particular parametric analysis is invalid.

For descriptive reference, theoretical min–max transformation places the reported knowledge mean at approximately 0.667 and the integration mean at approximately 0.733. These values locate each mean within its own response range. They do not demonstrate greater practice than knowledge, and the previously reported paired comparisons are not used as evidence of such a claim.

### 3.2. Correlations Among Constructs

Table 4 reports positive bivariate associations among the four self-report composites. The largest correlation is between integration and self-efficacy (r=0.812). Perceived knowledge correlates with integration (r=0.529) and self-efficacy (r=0.555); its correlation with attitudes is smaller (r=0.293). These associations do not establish temporal order, objectively demonstrated competence, or observed practice. In particular, the strong integration–self-efficacy correlation warrants investigation of overlapping item content and discriminant validity.

### 3.3. Faculty Profiles: Cluster Analysis

The reported silhouette coefficient favors k=2 (0.383) over k=3 (0.299), a decrease of 0.084. Three groups were retained for descriptive interpretability, a researcher-driven choice that does not follow the maximum-silhouette criterion (Figure 1). The two-group memberships and centroids are unavailable in the supplied materials, so no substantive comparison of the two partitions or stability claim is made. The decrease of 0.084 corresponds to approximately 21.9% of the two-group silhouette; this is a relative change in the reported criterion, not a statistical significance test. Calinski–Harabasz, Davies–Bouldin, gap-statistic, bootstrap-stability, and independent-replication results are unavailable and are not inferred from silhouette values. The present evidence does not establish three as the optimal number of groups. Table 5 retains the reported three-group means. Separation on these same clustering variables is expected by construction and is not independent evidence validating the profiles.

**Profile 1: Intermediate scores (39.6%; n=59).** This group has means between those of the other two groups on all four constructs: perceived knowledge 4.77/7, reported integration 3.79/5, self-efficacy 3.91/5, and attitudes 5.10/7. Weekly use is the most common reported frequency (52.5%). These results do not establish pragmatism or a lack of critical reflection.

**Profile 2: Higher scores (44.3%; n=66).** The largest group, although not a majority of the sample, has the highest means on all four constructs: perceived knowledge 5.69/7, reported integration 4.61/5, self-efficacy 4.60/5, and attitudes 6.45/7. Daily use is reported by 63.6%. Higher scores do not establish pedagogical effectiveness or use beyond demonstrated competence.

**Profile 3: Lower scores (16.1%; n=24).** This group has the lowest means on all four constructs: perceived knowledge 3.68/7, reported integration 2.41/5, self-efficacy 2.48/5, and attitudes 4.67/7. Women constitute 87.5% of this group. The label does not imply expertise without practice or a psychological barrier to adoption.

### 3.4. Variables Associated with the Profiles

Table 6 presents reconstructed counts, avoiding interpretation based only on percentages. The gender table reproduces the original statistic: $\chi^{2}\left(2\right)=4.840$, p=0.089, Cramér’s V=0.180. Thus, the numerical contrast in female representation does not establish a reliable gender difference at the nominal 0.05 threshold.

For the grouped frequency table, $\chi^{2}\left(4\right)=15.481$ and Cramér’s V=0.228. The conditional Monte Carlo probability is 0.0037, with Monte Carlo standard error approximately 0.00019. This supports an exploratory association in the grouped table while addressing its small expected cell. It is a different comparison from the source’s four-category test, $\chi^{2}\left(6\right)=18.80$, p=0.005; the latter cannot be reconstructed without separating occasional and single-trial counts by profile. Figure 2 displays the grouped percentages.

The original analysis also reports a training-recency association, $\chi^{2}\left(8\right)=24.83$, p=0.002, but overlapping labels and its 147-response denominator prevent a verified interpretation. No source association reaches the nominal 0.05 threshold for age (H=1.51, p=0.470), experience (H=0.53, p=0.766), qualification ($\chi^{2}\left(8\right)=6.55$, p=0.586), or modality ($\chi^{2}\left(4\right)=1.76$, p=0.780). Non-significance is not evidence of equivalence.

### 3.5. Hierarchical Regression: Adjusted Associations with Reported Integration

Table 7 presents the three steps of the hierarchical regression with pedagogical integration as the dependent variable.

The reported hierarchical models account for progressively larger proportions of variation in reported integration, with $R^{2}=0.196$ at Step 1, 0.381 at Step 2, and 0.677 at Step 3. Adding perceived knowledge at Step 2 increases $R^{2}$ by 0.185. Adding self-efficacy and attitudes jointly at Step 3 increases $R^{2}$ by a further 0.296; this increment cannot be assigned to self-efficacy alone. In the final model, self-efficacy has a positive adjusted association with reported integration (B=0.778, p<0.001). The coefficients for perceived knowledge (B=0.094, p=0.074) and attitudes (B=−0.028, p=0.557) do not reach the nominal 0.05 threshold. The reduction in the knowledge coefficient across steps describes a change in its conditional association after additional predictors enter the model. It does not establish mediation, the displacement of knowledge by confidence, or causal priority.

The source’s Step 1 statistic, F(4,141)=8.65, implies N=146 under ordinary regression with an intercept, whereas the main sample is 149. Also, $R^{2}=0.196$ and those degrees of freedom imply F≈8.59. No undocumented exclusions are inferred to resolve this discrepancy. The implied difference of three observations is not evidence that three participants were excluded for missing data: a reporting error is another possibility. Numbers excluded, reasons for exclusion, and missing-data handling at each step remain undocumented. The complementary model assumes a common complete correlation matrix with N=149; it does not perform or reconstruct participant-level deletion or imputation. The source reports VIF values below 2.70 and Durbin–Watson 1.846, neither of which establishes discriminant validity or rules out common-method bias.

### 3.6. Complementary Regression Reconstructed from Aggregate Correlations

A three-predictor model based on the reported four-construct matrix yields $R^{2}=0.6684$, adjusted $R^{2}=0.6615$, and F(3,145)≈97.43, conditional on the common-*N* and conventional OLS assumptions described above. Table 8 reports its coefficients. Self-efficacy has the largest standardized coefficient, β=0.758, with an approximate 95% interval [0.630, 0.885]. Perceived knowledge has a smaller coefficient, β=0.113, whose interval extends slightly below zero; attitudes have a near-zero estimate, β=−0.016.

Across the 64 evaluated rounding-corner matrices, $R^{2}$ ranges from 0.6674 to 0.6694, and the self-efficacy coefficient from 0.7554 to 0.7598. The knowledge coefficient ranges from 0.1116 to 0.1149, but its nominal *p*-value ranges from 0.0476 to 0.0540. Thus, a binary significance claim for knowledge is sensitive even to the precision of the reported correlations. The attitude coefficient ranges from −0.0179 to −0.0145. These ranges quantify rounding sensitivity only, not sampling or specification uncertainty (Figure 3).

The proximity of the complementary $R^{2}$ to the source’s final $R^{2}=0.677$ does not isolate the contribution of the omitted covariates: the analytic samples and models may differ. Similarly, agreement in the positive self-efficacy association is not an independent replication, since both presentations concern the same study and overlapping summary information.

## 4. Discussion

Taken together, the findings address the research questions at three levels. The composite correlations describe positive associations among perceived knowledge, reported integration, self-efficacy, and attitudes. The reported clustering summarizes an ordered pattern of scores, although the silhouette comparison favors two groups and does not validate the retained three-group partition. Finally, the regression results identify a positive adjusted association between self-efficacy and reported integration, conditional on the included predictors and the documented analytical limitations. These findings concern covariation among self-reports; they do not demonstrate objective competence, effective classroom implementation, or a causal pathway from confidence to practice.

### 4.1. Associations Without Cross-Construct Equivalence

The results describe covariation among self-reported perceptions and practices. They do not establish a direction of mismatch between objective knowledge and actual classroom behavior. Mapping the knowledge and integration scales onto identical endpoints changes their numerical presentation but does not calibrate their meaning. Consequently, the results do not substantiate an inverted knowledge–practice gap.

The three-group means show a broadly ordered pattern: the higher-scoring group has higher means on every construct, and the lower-scoring group has lower means throughout. This is compatible with a general gradient of reported engagement. The labels describe relative scores within this sample rather than discrete expertise levels or enduring personal characteristics; respondents close to a cluster boundary may be more similar than the categorical labels suggest. It does not, without further evidence, demonstrate qualitatively distinct psychological types. The intermediate group may offer a convenient description, but the stronger silhouette for two clusters challenges its interpretation as a separate underlying profile.

Rapid diffusion and easier access offer another possible context for the observed reports of use. In the sample, 133 of 149 respondents report daily or weekly GAI use (89.3%). This describes frequent reported engagement, but does not measure whether access barriers declined, whether everyday use preceded educational use, or how respondents’ understanding changed over time. A diffusion-based explanation is therefore a hypothesis for longitudinal investigation, not a demonstrated explanation of an “inverse paradox”. Such a paradox is not established by the present cross-construct scores. Repeated measurements of access, task-specific use, objectively assessed understanding, and perceived usefulness would help distinguish changing adoption conditions from stable individual differences.

More informative investigation of knowledge and practice would combine objectively scored conceptual tasks, observations or independently coded teaching artifacts, and self-report measures. Their relationships could then be studied without assuming that a common numeric range makes different constructs equivalent. Critical evaluation of outputs and the quality of assessment design would require their own measures.

### 4.2. Self-Efficacy and Shared Measurement Content

Self-efficacy has a strong bivariate association with reported integration and a positive adjusted coefficient in the reported final regression. However, the large correlation and similar item themes also make measurement overlap a plausible explanation for part of this association. A high alpha does not establish unidimensionality or demonstrate that integration and self-efficacy are empirically separable (Sijtsma, 2009). Shared method variance is a plausible source of association, not a bias quantified by this dataset (Podsakoff et al., 2003).

The non-significant final coefficient for perceived knowledge does not demonstrate that knowledge is irrelevant. Its interpretation depends on the other predictors, measurement error, shared variance, sample size, and model specification. Likewise, the attitudes coefficient does not establish that attitudes work through confidence. No temporal or indirect-effect analysis supports such a mechanism. Cross-sectional mediation can misrepresent longitudinal processes, providing a further reason to avoid inferring causal order here (Maxwell & Cole, 2007). The joint increase in explained variance when two predictors enter together is not an estimate of the unique contribution of either predictor.

These findings motivate examination of scale structure before theoretical claims are strengthened. An item-level evaluation should examine dimensionality and cross-construct overlap and report uncertainty. For example, an appropriate measurement-model analysis could evaluate the heterotrait–monotrait ratio, but this requires item-level associations and cannot be calculated from the four composite correlations (Henseler et al., 2015). Independent behavioral indicators would reduce reliance on a single response method. The exact model samples and frequency coding must also be recovered before the adjusted associations are treated as fully reproducible.

The complementary regression strengthens numerical transparency rather than causal interpretation. It shows that a large positive self-efficacy coefficient follows from the published correlation structure even without the demographic and frequency covariates. Its estimate remains similar at all examined rounding corners. In contrast, knowledge lies near the conventional significance threshold, so describing it as having either a definitively absent or a securely established adjusted association would overstate the available precision. The coefficient and its uncertainty are more informative than a threshold label.

### 4.3. Profile Characteristics and Gender

The lower-scoring group has a higher percentage of women than the other groups, but the sample is already predominantly female and the overall gender association is inconclusive (p=0.089). The small group size further limits precision. These data do not establish a gender-specific barrier, and profile composition should not be interpreted as evidence of a psychological mechanism.

Research on self-perceived digital competence and gender differences, such as the large-scale analysis by Navío-Inglés and colleagues, concerns broader measures and a different sample (Navío-Inglés et al., 2025). It identifies a relevant question for comparative research but cannot convert the present inconclusive association into evidence of a gender mechanism.

Reported use frequency differs across groups, as might be expected for groups partly defined by an integration scale. The conditional Monte Carlo analysis of grouped frequency avoids relying solely on an asymptotic approximation for its small expected cell. However, grouping loses the distinction between occasional and single-trial use and does not validate the original ordinal regression coding. Training recency also requires more careful reporting because the original labels overlap. Neither association establishes that training or frequent use produces membership in a particular group. Conversely, non-significant differences in age or experience do not justify declaring demographic factors irrelevant.

### 4.4. Limitations

The cross-sectional design cannot establish causal direction, mediation, or changes over time. All substantive constructs are self-reported, and no objective knowledge test, classroom observation, learning outcome, or independent evaluation of generated content is available. The study therefore cannot assess the accuracy, critical quality, or educational effectiveness of GAI use.

Measurement evidence is limited to the reported internal-consistency coefficients. The complete instrument and documentation of its development are needed to evaluate content coverage and scoring. Factorial and discriminant validity remain unestablished. In particular, the integration alpha of 0.981, the integration–self-efficacy correlation of 0.812, and shared item themes require explicit consideration of redundancy and common-method variance.

The sample is restricted to one faculty at one university. The reported response rate does not remove potential nonresponse bias or justify generalization to all Ecuadorian or Latin American faculty. The ten-month collection window may also combine responses from different stages of a rapidly changing technological environment; collection timing should be examined if respondent dates are available.

The three-cluster solution is researcher-selected and less well separated than the two-cluster solution according to the reported silhouette values. Its stability is unknown, the smallest group contains only 24 participants, and the original preprocessing description is ambiguous. Comparisons on clustering variables are circular as validation tests. Replication and sensitivity to preprocessing, initialization, and the number of clusters are needed before the groups can inform individualized policy.

Finally, the original model sample-size discrepancy, undocumented frequency coding, uncertain coefficient scale, and ambiguous training categories prevent complete verification of the original analyses. Complementary intervals and the reduced regression are derived from rounded summaries under explicit assumptions. They do not provide robust standard errors, residual checks, missing-data sensitivity, a two-cluster solution, or item-level validation. The deterministic rounding exercise measures only limited numerical sensitivity. Reconstructed count tests condition on the given cluster assignments and do not include uncertainty in how those groups were selected.

### 4.5. Practical Implications and Future Research

Faculty development can reasonably combine conceptual and critical AI literacy with supported opportunities to develop GAI-specific self-efficacy. Critical evaluation and informed educational use are also emphasized in discussions of AI literacy (Walter, 2024). Practice with feedback, evaluation of generated material, discussion of limitations, and pedagogical design may be complementary components. Their effectiveness and optimal sequence are questions for prospective evaluation; this observational study does not establish that confidence-building should replace or precede conceptual learning.

Higher reported integration does not identify unsafe or uncritical teaching. Concerns about unreliable outputs and dependence provide reasons to study critical evaluation, not findings about the present respondents (Kasneci et al., 2023). Training needs should therefore be assessed directly, including the quality of teaching artifacts and demonstrated understanding, rather than inferred from the difference between two normalized ratings.

Independent outcome measures are especially important when considering educational risks. Bastani and colleagues evaluate AI-supported mathematics practice and subsequent unassisted performance in a high-school experiment (Bastani et al., 2025). Their design illustrates why assisted activity and learning outcomes should be distinguished; it does not establish learning harm or uncritical use among the faculty surveyed here. The present dataset contains neither delayed learning assessments nor repeated measures of satisfaction, trust, or disappointment. It therefore cannot show that GAI compromises long-term learning or that faculty subsequently become disillusioned. Those possibilities should be evaluated with prospective student outcomes and repeated faculty assessments, rather than inferred from frequent use, favorable attitudes, or profile membership.

Institutional context also matters for the design of future studies. Ayanwale and colleagues examine teachers’ readiness and intention to teach AI in schools, while Falebita and Kok review strategic goals for AI integration among STEM academics and undergraduates in African higher education (Ayanwale et al., 2022; Falebita & Kok, 2024). These settings and objectives differ from university faculty’s reported GAI integration in Ecuador. Locally measured preparation needs and institutional conditions should therefore guide intervention design.

Institutions could use the descriptive results to formulate questions for needs assessment while avoiding fixed labels for individual faculty. Longitudinal or intervention studies with independent outcomes could evaluate whether combined conceptual, critical, and practical preparation is associated with improved educational use. Stable and externally validated profiles would be required before assigning faculty to different training pathways on the basis of clustering alone.

A practical research design would evaluate complementary training components without allocating people on the basis of unvalidated profile labels. One component could address conceptual interpretation of generated outputs; another could involve supported design and revision of teaching activities; a third could assess ethical and evidential judgment. Outcomes should include objectively scored knowledge tasks and independently evaluated teaching products, alongside the existing self-report domains. Such a design would test the proposed implications rather than treating them as demonstrated by correlations.

## 5. Conclusions

Among 149 faculty members from one Ecuadorian faculty of education, perceived conceptual knowledge of GAI, reported pedagogical integration, perceived self-efficacy, and attitudes show positive bivariate associations. The reported three-group partition describes comparatively higher, intermediate, and lower composite scores. However, the silhouette criterion favors two groups, and the available evidence does not establish a stable or externally validated classification of faculty.

Self-efficacy has a positive adjusted association with reported integration in the original hierarchical regression and in the complementary model reconstructed from aggregate correlations. The latter yields a standardized coefficient of approximately 0.758 under its stated assumptions. Shared item content and the absence of factorial and discriminant validity evidence limit the interpretation of this association as a relationship between empirically distinct constructs. The findings do not establish causality, mediation, or a deficit in knowledge relative to practice.

The results motivate prospective evaluation of faculty development that combines conceptual and critical AI literacy with supported opportunities to develop GAI-specific self-efficacy. Such evaluation should use validated instruments, objectively assessed knowledge, and independently evaluated teaching outcomes. The present study does not establish the effectiveness or optimal sequence of these training components, nor does it justify assigning faculty to training pathways on the basis of the reported clusters alone.

## Figures and Tables

**Figure 1:**
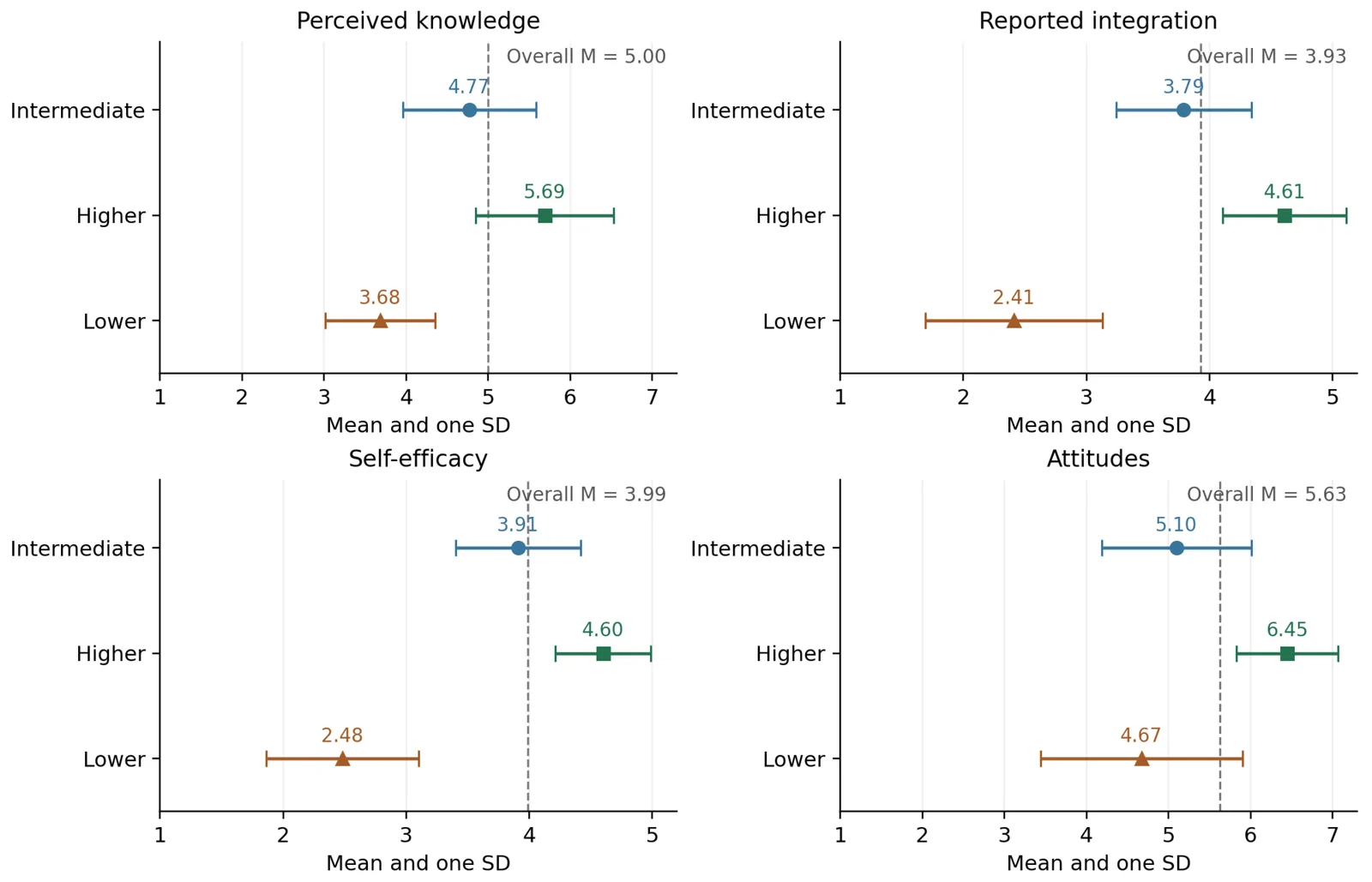
Reported means for the exploratory three-group partition. Circles, squares, and triangles mark the intermediate-, higher-, and lower-scoring profile means, respectively; numeric labels identify those means. Vertical dashed lines show the corresponding full-sample means (N=149). Horizontal bars show one reported within-profile standard deviation, not confidence intervals for estimated cluster membership. Panels use original score units. Comparisons across groups within a panel are descriptive; numerical heights across different constructs do not establish comparable mastery. Means are reproduced from Table 5.

**Figure 2:**
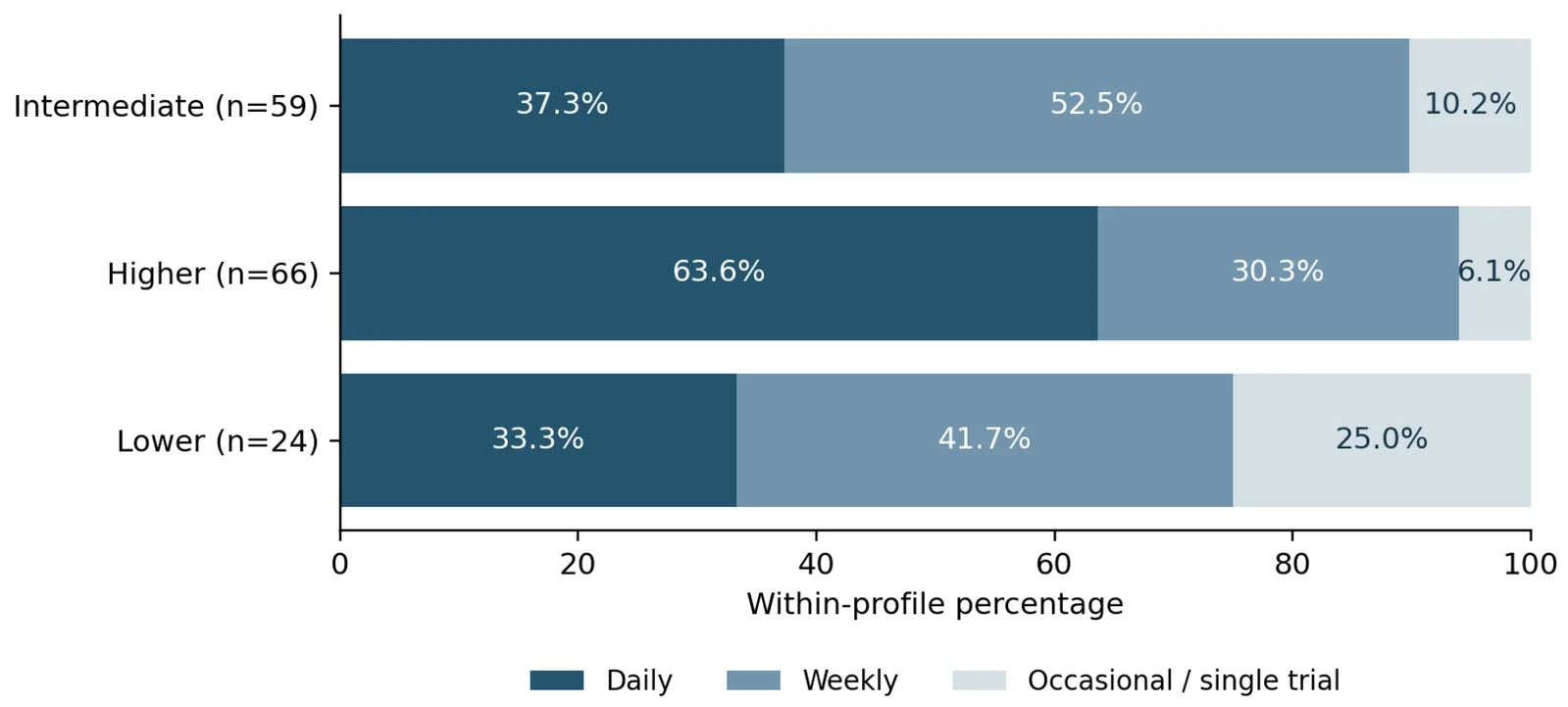
Frequency of reported GAI use within each exploratory profile. Percentages are calculated from the aggregate counts in Table 6. Occasional and single-trial use are combined; no respondent-level data are synthesized.

**Figure 3:**
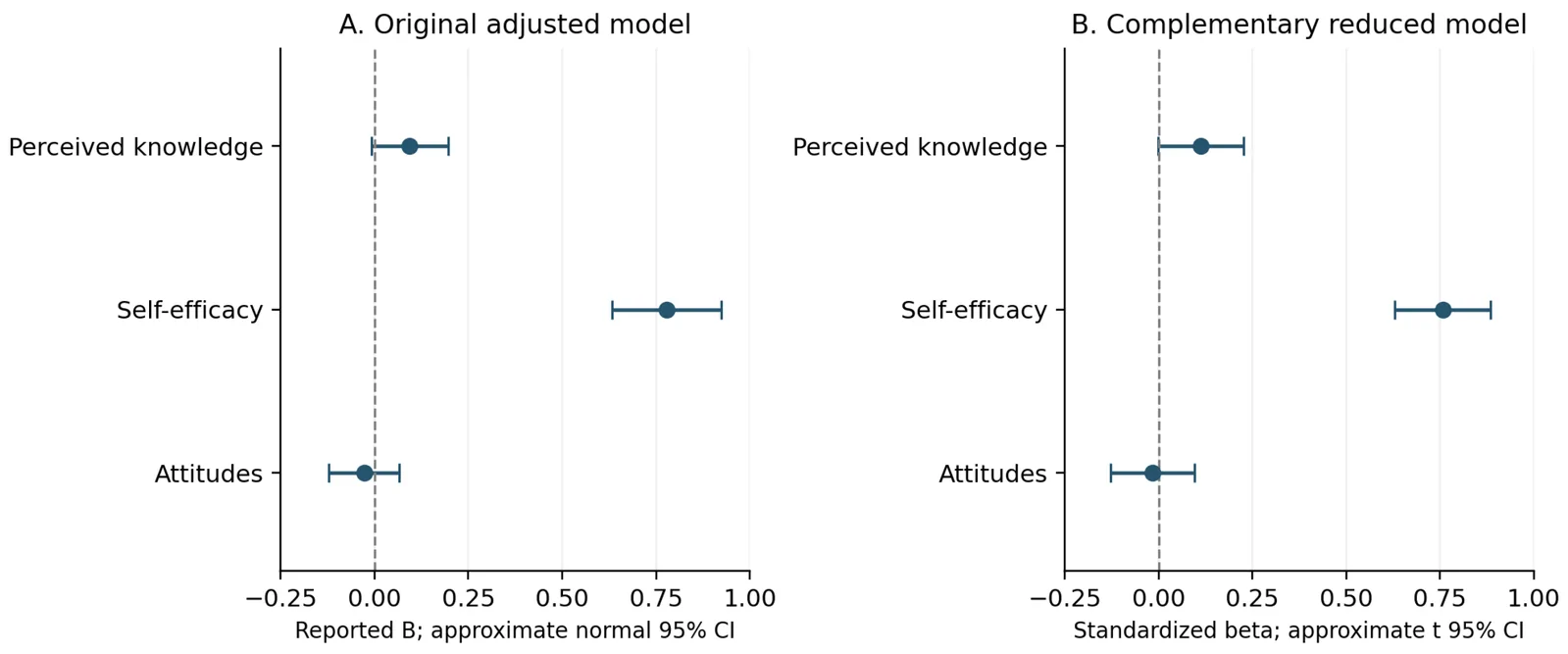
Coefficient estimates and approximate 95% intervals from distinct models. (**A**) uses source *B* coefficients from the original adjusted model and normal-reference intervals. (**B**) uses standardized coefficients reconstructed for the three-predictor model and *t*-reference intervals. Horizontal scales represent different coefficient units; magnitudes must not be compared directly across panels.

**Table 1:** Participant characteristics and reported GAI-use frequency.

Domain	Category	*n*	%
Gender	Women	105	70.5
	Men	44	29.5
Qualification	Master’s degree	103	69.1
	Doctorate	20	13.4
	Bachelor’s degree	22	14.8
	Postdoctoral qualification	2	1.3
	Specialization diploma	2	1.3
Teaching modality	Online	67	45.0
	In person	42	28.2
	Blended	23	15.4
	Not reported	17	11.4
GAI-use frequency	Daily	72	48.3
	Weekly	61	40.9
	Occasional	13	8.7
	Single trial	3	2.0

Note: All percentages use *N* = 149. Age: *M* = 42.5, *SD* = 10.1, range 24–65. Teaching experience: *M* = 14.1, *SD* = 10.0, range 1–48. Rounding may prevent percentages from summing to 100. Training recency has 147 tabulated responses and unresolved overlapping labels; it is not recoded here.

**Table 2:** Measurement scope and interpretation of the four self-report composites.

Construct	Items/Range	Reported Content Domains	Interpretation and Boundary
Perceived knowledge	12/1–7	Model operation; prompts; hallucinations and bias; tokens, context and temperature; ethical implications.	Perceived understanding; not an objectively scored knowledge test. Three reverse-worded items are reported.
Reported integration	15/1–5	Activity design, feedback, rubrics, differentiation, information verification, and responsible use.	Declared teaching-related practices; no independent assessment of implementation quality.
Self-efficacy	10/1–5	Confidence in troubleshooting, creating activities, supporting colleagues and students, and evaluating content.	Perceived capability; several activity domains overlap with integration.
Attitudes	8/1–7	Semantic-differential evaluations of GAI in the classroom.	Evaluative orientation; adjective pairs and exact anchors require the original instrument.

**Table 3:** Descriptive statistics and approximate mean confidence intervals (N=149).

Construct	Scale	Observed Range	*M*	SD	95% CI for *M*	α
Perceived knowledge	1–7	1.67–7.00	5.00	1.08	[4.825, 5.175]	0.841
Reported integration	1–5	1.00–5.00	3.93	0.95	[3.776, 4.084]	0.981
Self-efficacy	1–5	1.00–5.00	3.99	0.88	[3.848, 4.132]	0.947
Attitudes	1–7	2.25–7.00	5.63	1.13	[5.447, 5.813]	0.949

Note: Intervals are newly calculated from rounded reported summaries using a *t* reference with 148 degrees of freedom. They are model-based, not robust or finite-population-corrected intervals. Reported skewness/kurtosis: knowledge −0.315/0.284; integration −0.858/0.434; self-efficacy −0.996/0.814; attitudes −0.704/−0.057.

**Table 4:** Reported Pearson correlations with newly calculated approximate Fisher intervals (N=149).

Construct Pair	*r*	Approximate 95% CI
Perceived knowledge–Reported integration	0.529	[0.402, 0.636]
Perceived knowledge–Self-efficacy	0.555	[0.433, 0.657]
Perceived knowledge–Attitudes	0.293	[0.139, 0.433]
Reported integration–Self-efficacy	0.812	[0.749, 0.860]
Reported integration–Attitudes	0.414	[0.271, 0.539]
Self-efficacy–Attitudes	0.524	[0.397, 0.632]

Note: All source correlations are reported at p<0.001. Intervals use the Fisher transformation and rounded correlations; they do not establish discriminant validity or account for common-method bias.

**Table 5:** Descriptive characterization of the reported three-group partition (N=149). Values in parentheses are standard deviations.

Variable	Intermediate Scores	Higher Scores	Lower Scores
	(n=59, 39.6%)	(n=66, 44.3%)	(n=24, 16.1%)
Perceived knowledge (1–7)	4.77 (0.81)	5.69 (0.84)	3.68 (0.67)
Integration (1–5)	3.79 (0.55)	4.61 (0.50)	2.41 (0.72)
Self-efficacy (1–5)	3.91 (0.51)	4.60 (0.39)	2.48 (0.62)
Attitudes (1–7)	5.10 (0.91)	6.45 (0.62)	4.67 (1.23)
Women	71.2%	63.6%	87.5%
Daily GAI use	37.3%	63.6%	33.3%
Weekly GAI use	52.5%	30.3%	41.7%

**Table 6:** Profile characteristics reconstructed uniquely from rounded percentages and profile denominators.

Characteristic	Intermediate	Higher	Lower	Total
Women	42	42	21	105
Men	17	24	3	44
Daily GAI use	22	42	8	72
Weekly GAI use	31	20	10	61
Occasional/single trial	6	4	6	16
Profile total	59	66	24	149

Note: Gender and use-frequency blocks describe the same participants and must not be added together. The combined occasional/single-trial category is obtained by subtraction. No allocation of its 16 participants to the two original categories within profiles is assumed.

**Table 7:** Original hierarchical regression: reported coefficients and newly derived approximate normal-reference intervals.

Step	Predictor	*B*	SE	Source *p*	Approximate 95% CI
1	Age	−0.025	0.011	0.023	[−0.047,−0.003]
	Experience	0.029	0.012	0.014	[0.005,0.053]
	Gender (woman)	−0.209	0.156	0.182	[−0.515,0.097]
	Use frequency	0.481	0.100	<0.001	[0.285,0.677]
2	Age	−0.020	0.010	0.042	[−0.040,−0.000]
	Experience	0.017	0.010	0.096	[−0.003,0.037]
	Gender (woman)	−0.031	0.140	0.823	[−0.305,0.243]
	Use frequency	0.373	0.090	<0.001	[0.197,0.549]
	Perceived knowledge	0.398	0.061	<0.001	[0.278,0.518]
3	Age	−0.001	0.007	0.910	[−0.015,0.013]
	Experience	0.004	0.008	0.641	[−0.012,0.020]
	Gender (woman)	−0.040	0.102	0.699	[−0.240,0.160]
	Use frequency	0.164	0.068	0.018	[0.031,0.297]
	Perceived knowledge	0.094	0.052	0.074	[−0.008,0.196]
	Self-efficacy	0.778	0.074	<0.001	[0.633,0.923]
	Attitudes	−0.028	0.048	0.557	[−0.122,0.066]

Note: Reported $R^{2}$: Step 1 0.196; Step 2 0.381 ($\Delta R^{2}=0.185$); Step 3 0.677 ($\Delta R^{2}=0.296$). Intervals are B±1.96SE, calculated from rounded values; they are not exact model-specific *t* intervals. Source *p*-values are preserved. The coefficient scale, sample per step, and frequency coding require verification. These coefficients are not labeled standardized.

**Table 8:** Complementary three-predictor OLS model reconstructed from rounded aggregate statistics, assuming common N=149.

Predictor	*B*	β	Approximate 95% CI for β	Approx. *p*	VIF
Perceived knowledge	0.100	0.113	[−0.0004,0.2269]	0.0507	1.445
Self-efficacy	0.818	0.758	[0.6301,0.8852]	<0.001	1.821
Attitudes	−0.014	−0.016	[−0.1271,0.0948]	0.7737	1.379

Note: Outcome: reported integration. Predictors: perceived knowledge, self-efficacy, and attitudes only. *B* is derived using the reported sample standard deviations; β is standardized. Intervals use $t_{145}$ and conventional homoskedastic OLS errors. No demographic or frequency covariates are included. Four decimals are retained for the knowledge interval to show its small negative lower endpoint. This table is a new summary-statistic calculation, not recovered output from the original model.

## Data Availability

Participant-level data supporting the findings of this study are available from the corresponding authors upon reasonable request, subject to applicable confidentiality and ethical restrictions. The relevant aggregate data and analytical details are included in the article. These aggregate summaries contain no respondent-level records and do not reproduce the original clustering or seven-predictor regression.
